# Comparative SEM Study of Sensilla and Tyloid Structures in the Antennae of Vespinae (Hymenoptera: Vespidae)

**DOI:** 10.3390/insects15060448

**Published:** 2024-06-13

**Authors:** Tong Zhou, Xiaojuan Huang, Hasin Ullah, Yan Tang, Danyang Zhu, Hongli Xu, Qian Wen, Xiaoxia Tian, Jiangli Tan

**Affiliations:** Shaanxi Key Laboratory for Animal Conservation/Key Laboratory of Resource Biology and Biotechnology in Western China, Ministry of Education, College of Life Sciences, Northwest University, 229 North Taibai Road, Xi’an 710069, China; zhoutong@stumail.nwu.edu.cn (T.Z.); huangxiaojuan@stumail.nwu.edu.cn (X.H.); hasenullah888@yahoo.com (H.U.); tangyan1@stumail.nwu.edu.cn (Y.T.); zhudanyang@stumail.nwu.edu.cn (D.Z.); 202233084@stumail.nwu.edu.cn (H.X.); wenqian@stumail.nwu.edu.cn (Q.W.);

**Keywords:** antennal sensilla, distribution, morphology, scanning electron microscope, Vespinae

## Abstract

**Simple Summary:**

Our study examined antennal structures in nine species of Vespinae wasps, providing insights into their morphology and sensilla diversity. Using SEM, we identified 19 sensilla types, including unique variations in pit organs and sensilla trichodea. The males of seven species exhibited tyloids. This research enhances our understanding of sensory systems in Hymenoptera, aiding taxonomy and evolutionary studies.

**Abstract:**

This study investigates the distribution, morphology, and potential functions of antennal sensilla in various wasp species, including *Dolichovespula flora*, *D. intermedia*, *Vespula structor*, *Vl. vulgaris*, *Provespa barthelemyi*, *Vespa bicolor*, *V. ducalis*, *V. mocsaryana*, and *V. velutina* var. *nigothorax*. The study thoroughly analyzes the antennal structure of these species, representing all four genera of the yellow-jacket and hornet subfamily Vespinae. Using scanning electron microscopy (SEM), the study identifies a total of nineteen types of sensilla, including sensilla trichodea (ST-I, ST-II, ST-III), sensilla campaniform (SCF-I, SCF-II, SCF-III), pit organs (SCO-I, SCO-II, and SA), sensilla placodea (SP-I, SP-II), sensilla chaetica (SCH-I, SCH-II), sensilla basiconica (SB-I, SB-II), sensilla agmon (SAG-I, SAG-II), and sensilla coelocapitular (SCA). Additionally, tyloids were observed in the males of seven species, except for *Vl. structor* and *Vl. vulgaris*. The study provides insights into these sensilla types’ morphology, abundance, and distribution. It discusses the variations in sensilla morphology among different species and the presence of gender-specific sensilla. This study provides new data about the morphology and distribution patterns of sensilla and tyloid.

## 1. Introduction

Insects heavily rely on their sensory organs to navigate their environment and interact with conspecifics, prey, and the surrounding ecosystem [1,2]. Among these sensory organs, antennae are crucial in detecting and processing external stimuli, including odors, vibrations, and mechanical cues [3,4]. The antennae of wasps, belonging to the family Vespidae, are of particular interest due to their diverse ecological roles and complex behaviors [5,6]. Understanding the structure and function of antennal sensilla, the specialized sensory structures found on the surface of the antenna, is essential for unraveling the sensory capabilities and adaptations of wasps [7,8]. Antennal sensilla are known to mediate various sensory modalities, including olfaction, gustation, hygroreception, thermoreception, and mechanoreception [9,10]. By analyzing the distribution and morphology of sensilla, as well as their potential functions, we can gain valuable insights into the sensory ecology of wasps and their interactions with the environment.

Several studies have investigated the antennal sensilla of wasps, contributing to our understanding of their sensory adaptations. For instance, Mohamed et al. (2013) [11] conducted a study on the sensory structure and behavioral ecology of the Oriental hornet (*Vespa orientalis* L.) within the Vespidae family. Tan et al. [12] focused on the potentially lethal social wasps and the fauna of Chinese Vespinae. Carpenter and Kojima [13] compiled a species checklist in the Vespinae subfamily, providing valuable taxonomic information. Kimsey and Carpenter [14] explored the Vespinae species in North America, offering insights into their diversity and distribution. These studies have shed light on the diversity and distribution of antennal sensilla in different wasp species.

Other studies have examined the antennal sensilla of insects beyond the Vespidae family, building on the pioneering work in this field. For example, several studies by Schneider [15], Boeckh [16], and Altner et al. [17] laid the foundational understanding of insect antennal sensilla structure, function, morphology, and classification. Renthal et al. [18] more recently investigated the structure and distribution of antennal sensilla in the red imported fire ant, expanding on this earlier work. Similarly, Lambin et al. [19] studied the antennal movements of worker honeybees as indicators of odor detection, building on our knowledge of insect olfactory systems. Said et al. [20] analyzed the structure and function of the antennal sensilla of the palm weevil *Rhynchophorus palmarum*, further contributing to our understanding of sensory adaptations in other insect species. These foundational and more recent studies provide valuable insights into insects’ sensory capabilities and adaptations beyond the Vespidae family.

However, despite these valuable contributions, there is still much to learn about the antennal sensilla of wasps, particularly within the Vespidae family. This research manuscript aims to fill this knowledge gap by investigating antennal sensilla distribution, morphology, and potential functions in various wasp species. By employing scanning electron microscopy (SEM) and light microscopy, we examined the external structures of sensilla and their arrangements and densities along the antennae. Through careful observation and analysis, we identified and classified the different types of sensilla.

## 2. Material and Methods

The specimens were collected via hand sweeping. Most were from Shaanxi, besides *Dolichovespula intermedia* from Gansu and *Provespa barthelemyi* from Tibet, China. A total of 7 male and female individuals per species were observed on each species.

The antennae were carefully taken off from the antennal sockets using a pair of fine forceps under a stereomicroscope. For scanning electron microscopy, the antennae were fixed for 6 h in a 4% glutaraldehyde solution buffered with phosphate (pH 6.8), then dehydrated in a graded ethanol series (30%, 50%, 70%, 90%, 100%, 100%), replaced in isoamyl acetate twice for 30 min each time, dried in a CO_2_ critical-point dryer, sputter-coated with gold, and observed with a Hitachi S3400N scanning electron microscope at 15 kV. At least seven sensilla of each type were calculated, and their average and standard deviation sizes were derived using the software package SPSS Version 16 (http://www.spss.com) accessed on 20 August 2020.

Table 1 examines different types of sensilla in various wasp species. The mean values with standard deviations reported in Table 2 and Table 3 provide valuable insights into the size variations of sensilla among the different wasp species, enhancing our understanding of their sensory capabilities.

The morphological terminology follows Agmon et al. [21] and Mohamed et al. [11]. The voucher specimens were preserved in the College of Life Sciences, Northwest University, Xi’an, China (NWUX). A morphological cluster analysis was conducted on the antennal sensilla of the Vespinae species. The analysis utilized the cluster program Tree Analysis Technology (TNT). The dataset included sampling data for the studied taxa, and all morphological characteristics were treated as ordered. Each character state was assigned a specific numerical code to standardize the data. The data matrix (S 2) included multiple character states that represented the types and distributions of the sensilla based on their presence or absence in the specific taxonomic positions of the taxa. For the maximum parsimony (MP) analyses, new technology search algorithms were performed using TNT version 1.6. 

## 3. Results

### 3.1. General Morphology of the Antenna 

**Figure 1:**
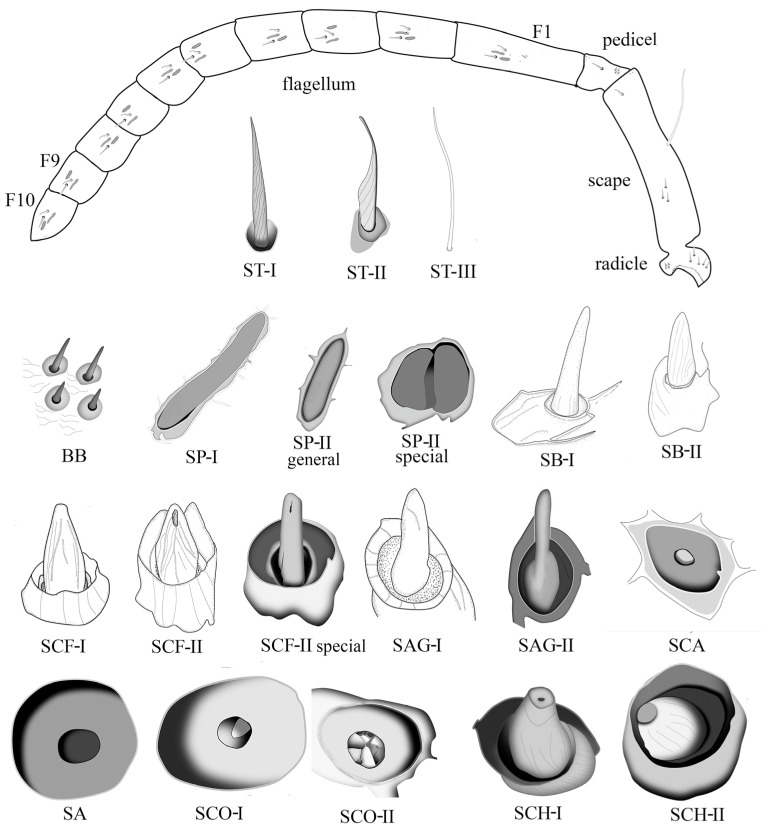
Antenna outline (F1 to F10 indicates the number of flagellomere) and different types of sensilla were observed in the Vespinae subfamily.

**Figure 2:**
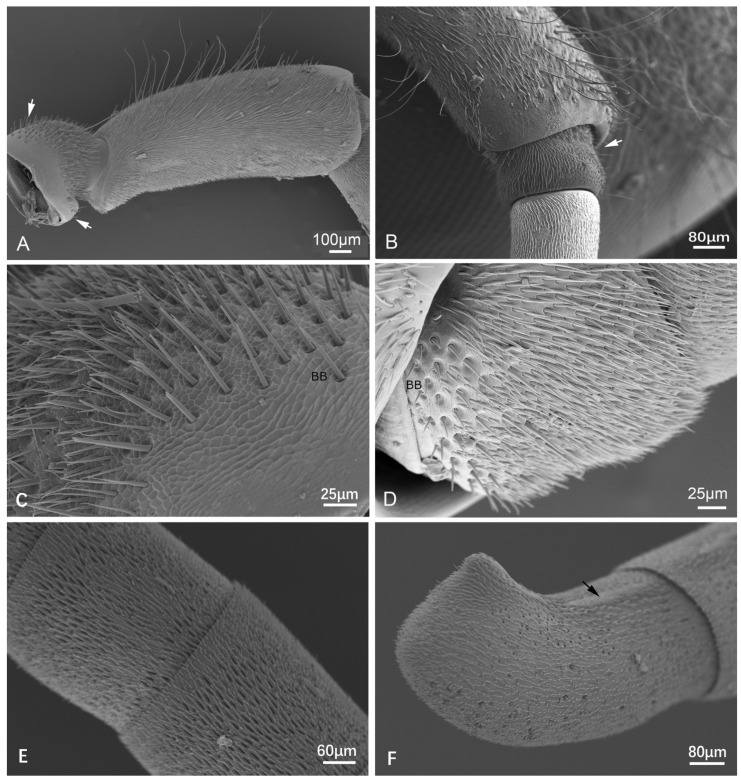
Scanning electron micrographs of antennal morphology and sensilla distribution. (**A**) the scape of male *Vespa velutina* var. *nigrithorax*, showing ST and BB on the basal radicle (white arrowed). (**B**) the pedicel of male *Dolichovespula flora*, showing many pores at the apical part and BB area (white arrowed). (**C**) the part of the basal radicle of male *V. velutina* var. *nigrithorax* amplified, showing BB and ST. (**D**) the pedicel of the female *Vespa bicolor*, showing BB and ST. (**E**) flagellomeres of female *Vespula structor*. (**F**) the terminal flagellomere of male *P. barthelemyi*, showing a truncated apex and a tyloid (black arrowed).

In general, the antennae of all the specimens are geniculate, consisting of three parts, namely the scape, pedicel, and flagellum (Figure 1). The scape is elongated with dense short setae and relatively sparse long setae (Figure 1 and Figure 2A,B), having a basal radicle that shows a scaly cuticle and dense setae fitting into the antennal socket (Figure 1 and Figure 2A). The pedicel is very short with scaly cuticles bearing numerous short setae and having an area with several pores apically (Figure 1 and Figure 2B). At the basal radicle of the scape and basal pedicel, there is an area with more than ten Böhm bristles (BB) clustered (Figure 1 and Figure 2C,D). The flagellum is long, consisting of 10 (female) or 11 (male) flagellomeres with various types of sensilla, as shown in the following part (Figure 1 and Figure 2E,F). The tip of the apical flagellomere is generally pointed (Figure 1), except for the male of *Provespa barthelemyi,* which truncates apically (Figure 2F). In the male, a few tyloids are located on the ventral flagellum, with the *Vespula structor* and *Vl. vulgaris* as an exception.

### 3.2. Different Types and Morphology of Sensilla with Distribution

There are 19 types of sensilla being observed on the antenna of both sexes (Figure 1), including three types of sensilla trichodea (ST-I, II, III), Böhm bristles (BB), two types of sensilla placodea (SP-I, II), sensilla campaniformia (SCF-I, II), sensilla chaetica (SCH-I, II), sensilla basiconica (SB-I, II), sensilla agmon (SAG-I, II), four types of pit organs (PO) showing as two types of sensilla coeloconica (SCO-I, II), the sensilla ampullacea (SA), and the sensilla coelocapitular (SCA). Additionally, many pores are observed on the scape and apical pedicel, and few pores are scattered on the flagellum. The sensilla types of each species have been listed in Table 1 and Figure 1.

Sensilla trichodea (ST), also known as trichoid sensilla, are believed to serve various sensory functions, such as sensory perception, mechanoreception, and detecting physical stimuli, such as air currents or vibrations in the environment. ST are long and slender, taper towards the tip, hair-like, bending along the antennal axis toward the apex with spiral furrows, and found on the whole antennae (Figure 1). ST are divided into three subtypes according to the length and shape, as follows. 

**Figure 3:**
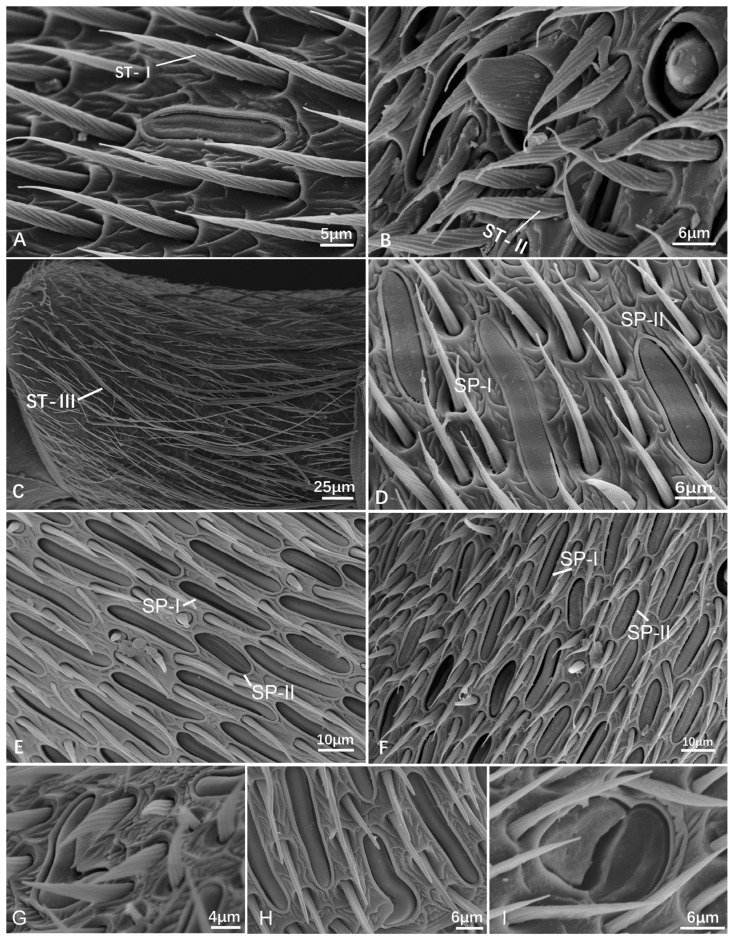
Scanning electron micrographs of the sensilla trichodea (ST) (**A**–**C**) and sensilla placodea (SP) (**D**–**I**). (**A**) Part of flagellum, female *Vespa velutina* var. *nigrithorax*. (**B**) Ibid, male *Vespa mocsaryana*. (**C**) Scape of female *Vespula structor.* (**D**) Female of *Provespa barthelemyi*. (**E**) Male of *Vespa mocsaryana*. (**F**) Female of *Vespa velutina* var. *nigrithorax*. (**G**–**I**) Showing special shaped SP: (**G**) a “L”-shaped SP-I, male *Dolichovespula flora*. (**H**) A hook L-shaped SP-I, male of *Vespa mocsaryana*. (**I**) A rounded bean-shaped SP-II, male of *Vespula structor*.

ST-I is mostly shorter and straighter than ST-II. The length is between 13.406 ± 0.951 μm and 25.607 ± 2.231 μm, the basal diameter is between 1.719 ± 0.113 μm and 2.754 ± 0.286 μm (Table 2). They are densely distributed, with the most common type being found in all studied species and being widespread on the scape, pedicel, and flagellomeres (Figure 1, Figure 2 and Figure 3A).

ST-II is similar to ST-I, but differs as its distal part being distinctly curved. The function is supposed to be the same as ST-I. Its size ranges from 12.57 ± 1.38 μm to 16.87 ± 1.56 μm long and 1.16 ± 0.15 μm to 1.56 ± 0.19 μm wide (Table 2). Their abundance became higher and higher towards the apical flagellum. ST-II was observed in all studied species. It is distinctly shorter and thinner than ST-I (Figure 1 and Figure 3B). It mostly bears on the flagellomeres.

ST-III is longer when compared to ST-I and ST-II, often with a pronounced curve or bend. They are present mostly in the scape. ST-III is characterized by its more elongated structure. In general, ST-III only bears on the scape (Figure 1, Figure 2A,B and Figure 3C). In a few species, ST-III is also on the last flagellomere, such as the female *Vl. structor* and *P. barthelemyi*, and the male *D. intermedia*. 

Böhm bristles (BB): These sensilla are similar to ST-I (Figure 2C) or SB-I (Figure 2D), with a socket depression present at the basal radicle of scape (12.23–63.19 μm long) and the basal pedicel (11.35–26.36 μm long). Normally, more than ten sensilla are clustered around one area and are without spiral furrows (Figure 2D). However, on the radicle of the scape, a few longer BB do have longitudinal furrows (Figure 2C). It is difficult to separate them from ST-I, except for the fact that the furrows are not spiral (Figure 1 and Figure 2C,D). BB is a mechanical sensor as a proprioceptor, being able to sense changes in the position of the antennae themselves.

Sensilla placodea (SP) are only present on the flagellum, are aligned in parallel with the antennal axis, and can be found in all studied species, being very common and in great quantity, shaped as an elongated boat, and with a flat plate embedded in the same shaped socket. The plate structure has three different shapes, namely flat, sunken, and raised (Figure 3D–F). In our studied species, among the female specimens, the flat plates predominate, while SP existed in males and are mainly raised or sunken plates. However, *Vespula structor* and *Vespa ducalis* show sunken plates both in males and females. They are divided into two subtypes based on the distinctly different ratios of length and width. Except for the general structure, three special types of SP are present in male *D. flora,* and *Vl. structor* was found first. For instance, an “L”-shaped SP-I (Figure 3G), a hook-like SP-I (Figure 3H), and a circle plate with a crack in the middle SP-II (Figure 1 and Figure 3I).

They are supposed to provide channels for volatile information substances and are generally believed to have an olfactory function. Some studies also suggest that it can sense infrared radiation, and is an important receptor for insects to perform long-distance chemical localization.

SP-I is relatively thin and long, being 21.630 ± 1.166 μm to 35.898 ± 2.117 μm long and 2.589 ± 0.247 μm to 4.600 ± 0.221 μm wide.

SP-II is relatively broad and short, being 15.26 ± 0.74 μm to 25.33 ± 0.38 μm long and 3.61 ± 0.27 μm to 4.87 ± 0.13 μm wide (Table 2). 

Sensilla basiconica (SB) are distributed along the entire length of the antennal flagellum but are sparsely found among the dense ST-I/ST-II and SP. It is a cone with a blunt top and with inconspicuous longitudinal furrows on the surface, without a distinct basal wall-like structure. Instead, the basal cycle socket is raised from the cuticle (Figure 1). The size changes from 8.33 ± 1.38 μm to 13.64 ± 1.04 μm in length and from 2.18 ± 0.17 μm to 3.49 ± 0.48 μm in basal socket diameter (Table 3). Sensilla basiconica can be further categorized into two subtypes according to size. 

**Figure 4:**
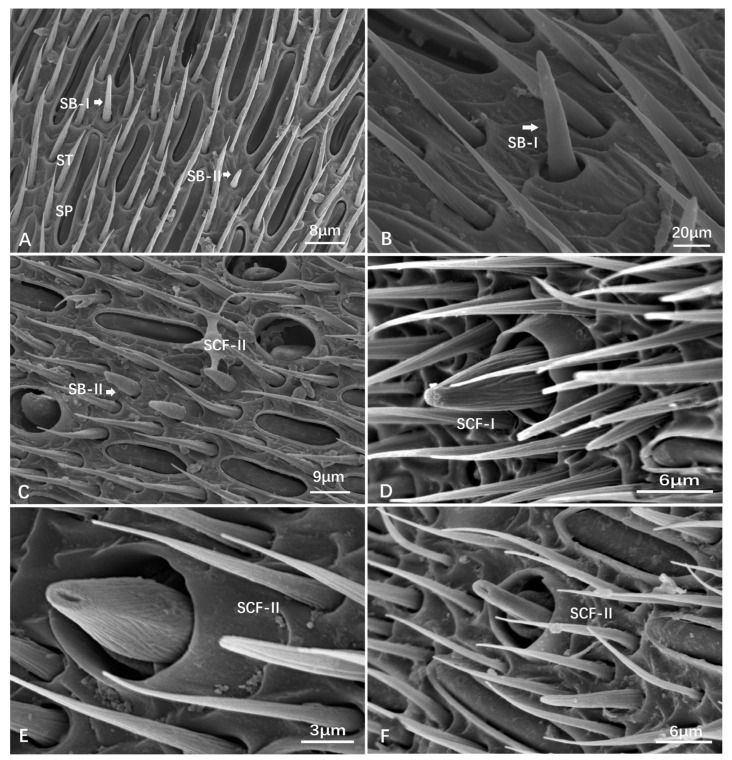
Scanning electron micrographs of the sensilla basiconica (SB) and sensilla campaniformia (SCF). (**A**) Female *Vespula structor*. (**B**) Female *Provespa barthelemyi*. (**C**) Female *Vespa ducalis*. (**D**) Male *D. flora*. (**E**) F8, male *Vespula structor*. (**F**) F9, ibid, female.

SB-I is typically longer and thicker when compared to SB-II sensilla (Figure 4A,B). 

SB-II is shorter and thinner than SB-I sensilla (Figure 4A,C).

Sensilla Campaniformia (SCF) are dome-shaped structures with a central depression. They have a relatively big or high basal annulus-shaped wall structure and a stout volume cone with longitudinal furrows on the surface. The height of the cone (*n* = 7) varies from 9.09 ± 0.37 μm to 12.47 ± 1.65 μm, and the inner diameter of the wall structure ranges from 6.11 ± 0.47 μm to 9.19 ± 0.59 μm (Table 3). According to whether the aperture is on the top or not, the SCF can be further categorized into two subtypes as follows:

SCF-I, which looks like a solid cone without an aperture on the top (Figure 4D);

SCF-II, which looks like a hollow cone with an opening on the top (Figure 4E). Additionally, a special type of SCF-II was observed first, which is a column-like structure with a top opening (Figure 4F).

SCF is generally distributed on the flagellum. In *Provespa barthelemyi*, SCF-I is found on F6, and specialized SCF-II is found in females, while a small number of SCF are distributed on F11 in males. A unique SCF-II is observed on F9 in the female *Vespula structor*, and many SCF-II are present on F11 in male *Vespa mocsaryana*. In *Vespa bicolor*, SCF-II is found on F8 in females and F4 in males. A large number of SCF-I are distributed on the distal flagellomeres in female *Vespa ducalis*, with various sensilla, including a large number of SCF-II, on the tip of F11 in males. In female *Vespa velutina* var. *nigrithorax*, SCF-II is found on F4, and both SCF-I and SCF-II are on F9.

**Figure 5:**
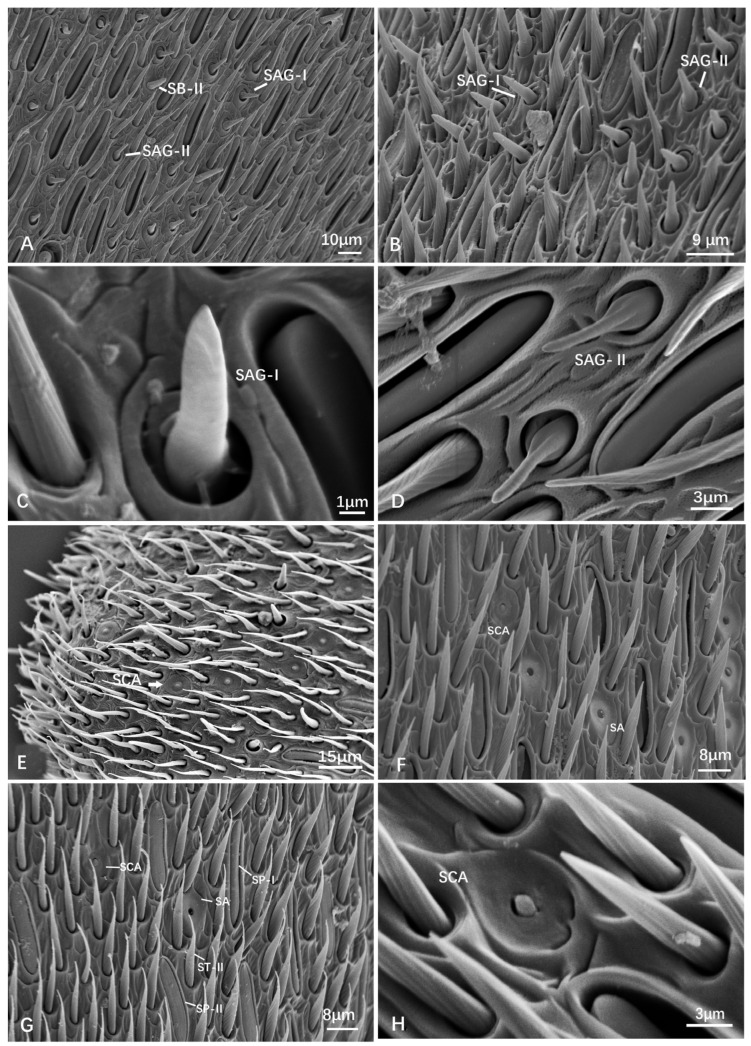
Scanning electron micrographs of the agmon (SAG) and sensilla coelocapitular (SCA). (**A**) F1, female *Vespa ducalis*. (**B**) Female *Vespula structor*. (**C**) Male *Dolichovespula intermedia*. (**D**) Male *Vespa mocsaryana*. (**E**) Apical F11, male *Provespa barthelemyi*, showing SCA clusters. (**F**) F4, male *Vespa velutina* var. *nigrithorax*. (**G**) F4, female *Vespa bicolor*. (**H**) F11, male *Dolichovespula intermedia*.

Agmon sensilla (SAG) exhibits a structure with a blunt top fitting into a thick annulus-shaped wall rising above the cuticle, smooth and lacking any obvious grooves. They are columnar in most of the species, except for male *Dolichovespula intermedia* and female *Vespa mocsaryana*. The female *Vespula structor* are special as we cannot see the obviously big bottom. Sometimes, SAG appear together and form a cluster. The plate ranges from 6.27 ± 0.61 μm to 10.46 ± 0.13 μm in length and the inner diameter of the wall is from 2.43 ± 0.14 μm to 3.98 ± 0.25 μm (Table 3). According to the shape, SAG can be divided into two subtypes as follows:

SAG-I: Small and conical sensilla with a slightly more rounded tip and a narrower base insertion into a shallow pit. Their surface texture is completely smooth, lacking visible microstructures. The embedded structure presents a big bottom and a thinner upper part; they are columnar (Figure 5A–C). 

SAG-II: Small and conical sensilla with a slightly more pointed tip and a base insertion into a shallow pit with a broader base. Their surface texture is smooth, but may have faint microgrooves (Figure 5A,B,D).

SAG is generally present on the flagellum. In *Dolichovespula intermedia* and female *D. flora*, SAG-I is distributed on the proximal flagellum. In female *Provespa barthelemyi* and female *Vespula vulgaris*, SAG-I is observed on F4 and F6. Female *Vespula structor* has SAG-II on F5, while female *Vespa bicolor* has SAG-I on F4. Both SAG-I and SAG-II are distributed along the flagellum in female *Vespa ducalis*, with many SAG-II found on the F7 of males. In female *Vespa velutina* var. *nigrithorax*, SAG-I is present on F7. They were distributed on the segments of the antennae, and their density gradually increased toward the apex.

**Figure 6:**
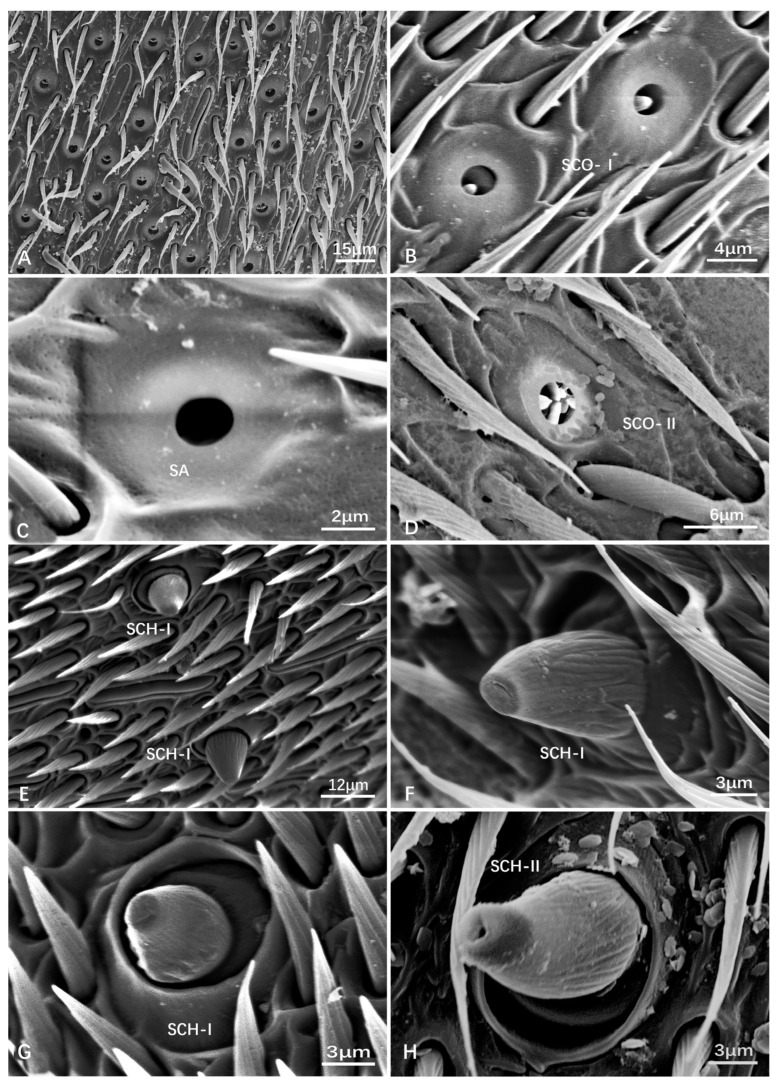
Scanning electron micrographs of the pit organs (PO) (**A**–**D**) and sensilla chaetica (SCH) (**E**–**H**). (**A**) a cluster of PO (SA, SCA, and SCO) on female *Provespa barthelemyi.* (**B**) female *Dolichovespula intermedia*. (**C**) male *D. flora*. (**D**) male *Vespa velutina* var. *nigrithorax*. (**E**) male *Dolichovespula flora*. (**F**) female *V. mocsaryana*. (**G**) female *D. intermedia*. (**H**) female *Provespa barthelemyi*.

Pit organs (POs) have various shapes, including peg-like or hair-like structures inner side. They have a sensory pore or opening at the tip. The diameter of the bulbous structure was measured and listed in Table 3. Additionally, a special pit organ was found in male V. *velutina* var. *nigrithorax*, showing several raised structures around the hole (Figure 6D). There is no obvious size difference between these types. The diameter of the inner hole is from 2.23 ± 0.29 μm to 4.08 ± 0.25 μm. POs are further divided into sensilla coelocapitular (SCA), sensilla coeloconica (SCO-I, II), and sensilla ampullacea (SA). These pit organs serve as thermoreceptors, aiding the insects in regulating their body temperature, locating heat sources, or responding to environmental temperature changes.

Sensilla coelocapitular (SCA) are button-shaped sensilla (Figure 5E–H). SCA are generally rare compared to other types; however, they could be gathered on the tip of terminal flagellomere (F10/F11). For instance, quite a few SCA were observed on the F11 of male *Provespa barthelemyi* (Figure 5E). Additionally, in *Dolichovespula intermedia*, the concavity is heart shaped rather than circular (Figure 5H).

Sensilla ampullaceal (SA) has a completely hollow structure, a bulbous or flask-like shape, and a swollen base, known as the ampulla, which connects to the cuticle of the antenna. From the ampulla, a narrower sensory shaft extends outward.

Sensilla coeloconica (SCO) appears as cuticular openings. SCO-I appears with a peg-like protrude in the center (Figure 6B). SCO-II is special, with many peg-like protrudes in the center. Among the checking species of Vespinae, only one SCO-II was found in the F8 of male *Vespa velutinae* var. *nigrithorax* (Figure 6D). However, many SCO-II were found in the F10 of male *Eustenogaster micans* (Vespidae: Stenogasterinae) (unpublished).

SCO and SA are distributed on the flagellum. For instance, in *Vespa bicolor*, SCO-I is observed on F5 (Figure 5G), while, in male *V. velutina* var. *nigrithorax*, SCO-II is found on F8 (Figure 6D). SCO-I and SA are found on F4 (Figure 5F). Across all of the nine studied species, SCO and SA are prevalent types of sensilla, highlighting their commonality and potential significance in sensory functions. 

Sensilla chaetica (SCH) consists of a short wall structure and stocky cone with irregular longitudinal striations on its surface, similar to SCF in terms of the cone shape protruding from the socket. The bottom of SCH can be seen from a dorsal view, while that of SCF cannot be observed. Most of the cones then shaped truncated cones (Figure 6E–H). SCH can be further categorized into two subtypes as follows:

SCH-I truncates apically without opening (Figure 6E–G). SCH-I is primarily mechanoreceptive and plays a role in detecting movement and tactile stimuli.

SCH-II with an opening on the tip (Figure 6H). SCH-II is believed to have a dual function, serving both mechanoreceptive and chemoreceptive roles. They may have structures such as pores that allow them to detect chemical stimuli.

SCH was observed on the flagellum. In female *Provespa barthelemyi*, SCH-I and SCH-II are observed on F10. In female *Vespula vulgaris*, SCH-I is found on F3 and F5. In the female *Vespula structor*, SCH-II is present on F6. In female *V. bicolor*, SCH-I is found on F4, F7, and F8. A special SCH-II was found in female *Provespa barthelemyi*, with a crateriform opening on the top of the cone (Figure 6D). These are a less distributed type of sensilla, occurring more in females than in males (Table 1).

### 3.3. Tyloids 

Tyloids are only present on the ventral surface of the male flagellum. They are specialized, small, elongated, coarse, and irregular keratinized areas that protrude from the surface of the antenna with many small holes. They are believed to be a structure of cuticular glands and to be involved in antennal coils, in transferring pheromones secreted by glands, and in courtship behavior, not in sensory organ clusters. They were found on the inner side of flagella in males, except for *Vespula structor* and *Vespula vulgaris,* within the nine species. The amount is different in the different species. In most species, there is one tyloid on the first flagellomere (F1) and two in the others, with a few exceptions. There are two tyloids on the F1 of *Vespa mocsaryana,* while there is no tyloid on the FI of *V. ducalis*. In *Dolichovespula intermedia*, the first tyloid was found in the F8, with each then following flagellomere with two tyloids, except for only one tyloid being present on F11 (Table 4). 

**Figure 7:**
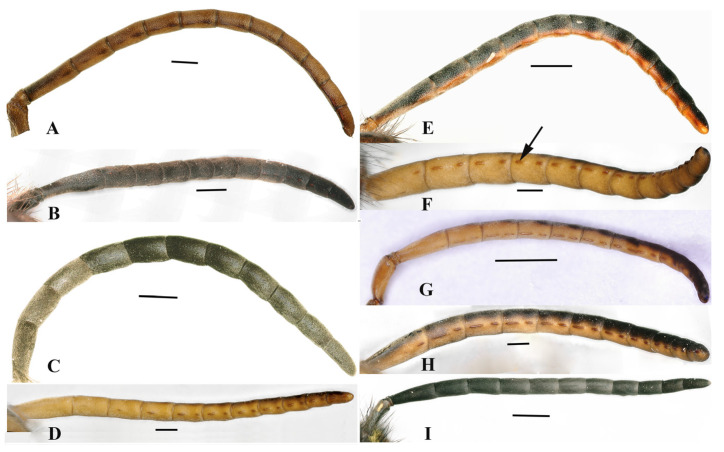
Light micrography of tyloids on the male antennae. The arrow represents tyloids. (**A**) *Provespa barthelemyi*. (**B**) *Dolichovespula intermedia*. (**C**) *Vespula structor*. (**D**) *Vespa ducalis*. (**E**) *Dolichovespula flora*. (**F**) *Vespa velutina* var. *nigrithorax*. (**G**) *Vespa mocsaryana*. (**H**) *Vespa bicolor*. (**I**) *Vespula vulgaris*. Scale bar = 500 μm.

**Figure 8:**
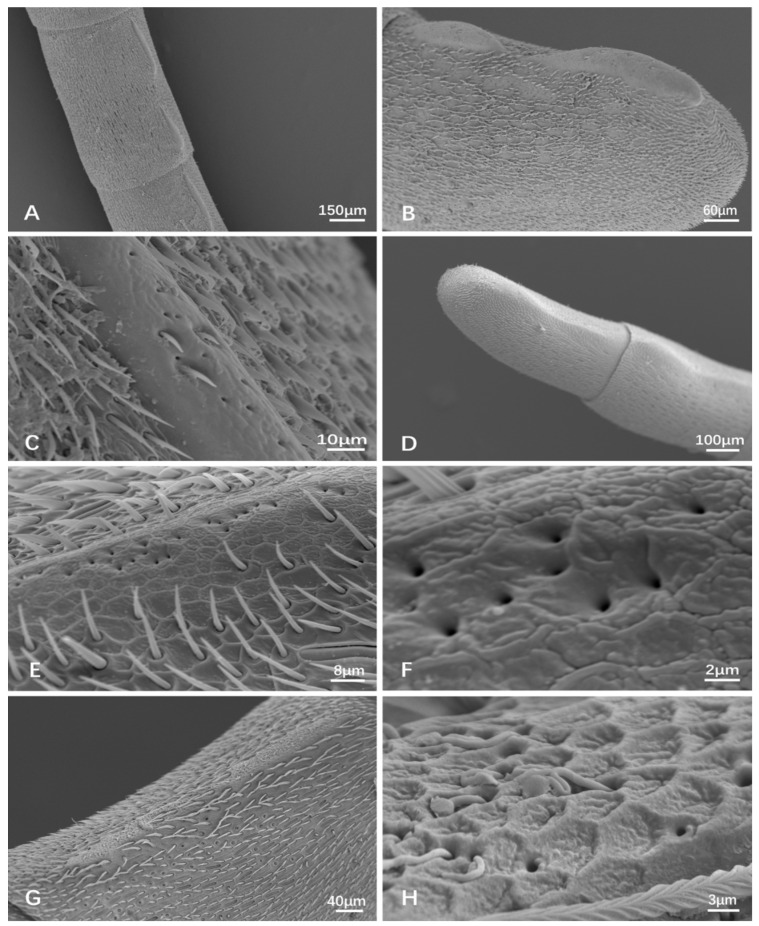
Tyloids on the male antennae, SEM. (**A**–**C**) *Vespa velutina* var. *nigrithorax*. (**D**–**F**) *Dolichovespula flora*. (**F**) Ibid, showing pores on the tyloid. (**G**,**H**) *Provespa barthelemyi*.

On the whole, there are two display modes of tyloids, viz. two tyloids being separate on one flagellomere (Figure 7B,D,F–H), or two tyloids being connected into one (Figure 7A,E). The ultrastructure of the separate tyloid types shows relatively smooth cuticula, with several pores and with few short and sparse setae (Figure 8A–C); the other type shows rough reticulate cuticula with pores (Figure 8D–H).

*Vespula structor* and *Vespula vulgaris* do not show the presence of tyloids (Figure 7C,I). *Dolichovespula intermedia* have very indistinct separated tyloids with two on F8, F9, and F10, and only one on F11 (Figure 7B). *Dolichovespula flora*, *Vespa bicolor*, *Vespa velutina* var. *nigrithorax*, and *Provespa barthelemyi* show the same tyloid numbers for each flagellomere (Figure 7A,E,F,H). *Vespa ducalis* displays little difference. F1 showed zero, F2, F3, and F4 showed one, and the remaining flagellomere showed two tyloids on each (Figure 7D). *Vespa mocsaryana* showed two tyloids on each flagellomere (7G). 

### 3.4. Cluster Analysis Based on Antennal Sensilla and Tyloids

**Figure 9:**
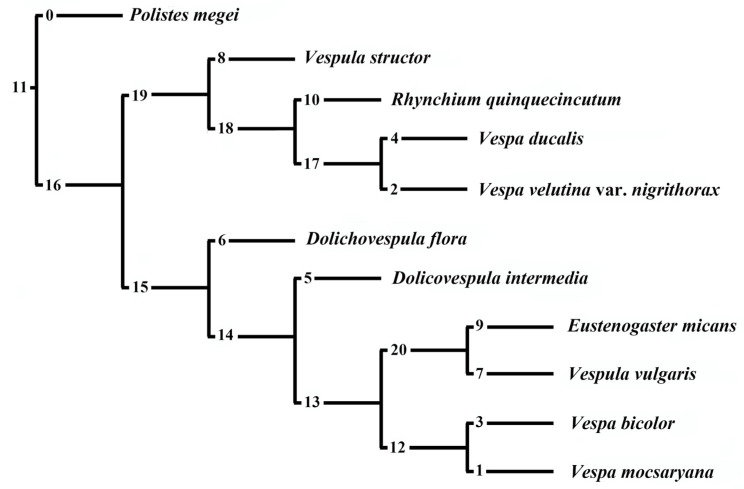
Cluster analysis based on antennal sensilla and tyloids. The digits on the branches indicate the total number of character changes (steps) along that branch.

Based on the morphological data of the antennal sensilla of the eight vespids species (this study) and three species (unpublish data in our lab) representing all four subfamilies of Vespidae from China as outgroups, we used TNT for cluster analysis. The ordering of the sensilla characteristics is provided in Table S1, and the Matrix characteristics of the antennae used for the phylogenetic tree are provided in Table S2. The cluster results show that the antenna of *Polistes megei* is most different from all others. The studied species were separated into two groups (Figure 9) as follows: (1) *Vespa ducalis* sibling to *Vespa velutina* var. *nigrithorax*, their two combined with *Rhynchium quinquecinctum,* similar to *Vespula structor*; (2) *Vespa bicolor* is similar to *Vespa mocsaryana*, *Vespula vulgaris* is similar to *Eustenogaster micans*, and these four species combined with *Dolichovespula intermedia* are siblings to *Dolichovespula flora*. Surprisingly, the results poorly supported their taxonomic taxon (genus). More research is expected to understand the character’s utility for phylogeny analysis.

## 4. Discussion

Insects are susceptible to a wide range of mechanical stimuli, including touch, sound, air currents, and body area deformations brought on by either self-generated movement or external forces [22,23]. Sensilla trichoidea (ST-I, ST-II, ST-III), sensilla placodea (SP-I, SP-II), sensilla chaetica (SCH-I, SCH-II), sensilla campaniformia (SCA), and sensilla basiconica (SB-I) are currently thought to be in charge of mechanoreception in Gerromorpha, according to the morphological study [24]. The current study describes several structures, with a specific focus on SP-I, SP-II, SCF-II, SCH-II, and SCA, which were notable findings in this study. 

We observed that sensilla placodea (SP) are aligned in parallel with the antennal axis and can be found in all studied species. They consist of a plate structure and are divided into two types based on the distinctly different ratios of length and width. Additionally, our investigation revealed significant variation in the shape of SP among different insect families. For instance, in the Sphecidae family, SP appeared as a round chassis with an irregular convex structure in the middle, which may suggest adaptations for specific environmental interactions or sensory functions unique to this family [25,26]. In contrast, in the Braconidae family, the plates are extremely long and obtuse at both ends, potentially reflecting a different mode of sensory detection or structural requirements [27,28]. Similarly, in the Vespidae family, SP exhibited longitudinal shapes that were obtuse at both ends, indicating a possible adaptation for their ecological niche or behavioral traits. Conversely, in the Apidae family, SP took the form of a disc with a central depression, which might be associated with their distinct foraging behaviors and communication methods [29,30]. These variations underscore the diverse evolutionary adaptations of SP across different insect families, highlighting the importance of shape and structure in their respective sensory functions. Comparing these morphological differences with the results from various authors provides a comprehensive understanding of how SP have evolved to meet the specific needs of each family. These variations in shape were consistently observed across different taxonomic groups, highlighting the notable differences in SP morphology.

We reported a special SCH in female *Provespa barthelemyi*, namely a crateriform opening on the top of the cone (Figure 6H). They are a less distributed type of sensilla, occurring more in females than in males. Mohamed et al. [11] reported that *Vespa orientalis* (SCH) only exists in females, and the type SCF II (Figure 4E) is mere and only in males. However, in our study, SCH was also found in the males of *Vespula structor*, *Dolichovespula flora*, and *Vespa ducalis*; and several SCF II were observed in the females of *Vespa ducalis* and *Vespa velutina* var. *nigrithorax*. 

We observed PO sensilla, characterized by irregular circular concavity with a bulbous or fastener-like structure, often present on the apical flagellomere. This sensilla type was abundant across most species, except in *Dolichovespula intermedia*, where heart-shaped concavities were occasionally seen (Figure 5H). The size of the bulbous structure was measured (Table 3). Interestingly, while Polidori et al. [31] reported the absence of a similar sensilla type (SCA) in the females of certain bee species, our study found SCA in both sexes of the studied wasp species. This suggests potential differences in the sensilla presence between bees and wasps. Additionally, Gerónimo et al. [32] found a higher abundance of sensilla-type ST-II in the females of Emphorini bees, but our study observed either a scarcity or absence of ST-II in certain female wasps, like *Dolichovespula flora* and *Vespa bicolor*. These variations in sensilla presence and abundance among species and sexes underscore the need for further comparative studies across a broader range of wasp species. 

The phylogenetic relationships within the eusocial subfamily Vespinae remain controversial based on morphological behavioral and/or molecular data up to now [33,34]. More data are expected to help solve the problem. Therefore, we compared the size and morphological data of each type of sensilla and tried to analyze the kinship among the nine species. The results showed that sensilla placodea (SP) and tyloid may act as special characters for Vespidae. They are distinctly different from Andienidae (circular or oval plates), Apoidea (flat discs), Nyssoninae, and Philanthinae (the plates, situated in craters, are raised as low pegs) (Hymenoptera, Sphecidae) [25,31,35,36]. Tyloid has long been used in phylogenetic studies [37,38]. In our study, the conclusion of kinship based on the results of tyloid may be consistent with the conclusions of previous studies to some degree (Table 4). For instance, Perrard et al. [38] suggested that *Vespa velutina* var. *nigrithorax* was closer to *Vespa bicolor* than to its other congeners based on morphological and molecular data. Lopez-Osorio et al. [39] mentioned that the genus *Dolichovespula* was more closely related to *Vespa* than to *Vespula* via analyzing transcriptomic (RNA-seq) data. Our results on tyloids do not support their opinions. However, it disagreed with Perrard et al. [38] on the genus *Provespa* being closer to *Dolichovespula* than to *Vespa*. Furthermore, it does not align with the findings of Fan et al. [40] and Wang et al. [34], who all support the clade (*Vespa* + *Vespula*) being a sister group to *Dolichovespula*. In our tyloids observation, *Dolichovespula* is closer to *Vespula* than to *Vespa* (Table 4). Obviously, as a morphological character, the tyloid could play a limited role in phylogeny. Future research should explore the functional significance of these sensilla types and their roles in species-specific behaviors and ecological adaptations within the Vespidae family. By conducting comprehensive studies, we can deepen our understanding of sensory adaptations and their implications in the biology of wasps.

## Figures and Tables

**Table 1:** Different types of sensilla are examined in various species.

Species	Gender	ST-			SP-		SCF-		SCH-		SAG-		SB-		SA	SCO-		SCA
		I	II	III	I	II	I	II	I	II	I	II	I	II		I	II	
*Dolichovespula intermedia*	♀	+	+	+	+	+	−	−	−	+	+	−	+	+	+	+	−	+
	♂	+	+	+	+	+	−	+	+	−	−	+	+	−	+	−	−	+
*Dolichovespula flora*	♀	+	−	+	+	+	+	+	+	+	+	−	+	−	−	−	−	+
	♂	+	+	+	+	+	+	+	+	−	+	−	+	+	+	+	−	−
*Provespa barthelemyi*	♀	+	+	+	+	+	+	+	+	+	+	−	+	−	+	+	+	+
	♂	+	+	+	+	+	+	+	−	−	−	−	+	+	+	+	−	+
*Vespula vulgaris*	♀	+	+	+	+	+	+	−	+	−	+	−	−	−	+	−	−	−
	♂	+	+	+	+	+	+	−	+	−	−	−	+	−	+	−	−	−
*Vespula structor*	♀	+	+	+	+	+	+	+	−	+	+	+	+	+	+	−	−	+
	♂	+	+	+	+	+	+	+	−	−	+	+	+	+	+	−	−	+
*Vespa mocsaryana*	♀	+	+	+	+	+	−	−	+	+	−	−	−	+	+	+	−	+
	♂	+	+	+	+	+	−	+	−	−	+	−	+	+	+	−	−	−
*Vespa bicolor*	♀	+	+	+	+	+	−	+	+	−	+	−	+	−	+	−	−	+
	♂	+	+	+	+	+	−	+	−	−	−	−	+	+	+	+	−	+
*Vespa ducalis*	♀	+	+	+	+	+	+	+	−	−	+	+	+	+	−	−	−	−
	♂	+	+	+	+	+	−	+	+	−	+	+	+	+	+	−	−	−
*Vespa velutina* var. *nigrithorax*	♀	+	+	+	+	+	−	+	+	−	+	+	+	−	+	−	−	−
	♂	+	+	+	+	+	−	+	+	+	+	+	+	+	+	−	+	+

Notes: “+” indicates the presence of the sensillum; “−” indicates the absence of the sensillum.

**Table 2:** The size of ST and SP on antennae in the males and females of nine species (*n* = 7).

	Sensilla	ST-I		ST-II		SP-I		SP-II	
Species		Basal Diameter	Length	Basal Diameter	Length	Width	Length	Width	Length
*Vespula structor*	M	2.186 ± 0.177	19.914 ± 1.489	1.25 ± 0.09	14.20 ± 0.83	3.118 ± 1.332	28.460 ± 5.295	4.456 ± 0.378	17.707 ± 0.722
	F	1.965 ± 0.160	13.682 ± 1.120	1.16 ± 0.15	12.57 ± 1.38	2.926 ± 0.485	24.202 ± 0.605	3.306 ± 0.387	17.667 ± 0.260
*Dolichovespula intermedia*	M	2.002 ± 0.129	14.855 ± 0.951	1.80	14.4	2.86 ± 0.18	28.725 ± 0.949	3.90 ± 0.13	15.26 ± 0.74
	F	1.719 ± 0.113	13.406 ± 1.49	1.71	8.19	3.192 ± 0.277	26.520 ± 1.233	4.041 ± 0.542	18.219 ± 0.972
*Dolichovespula flora*	M	2.145 ± 0.156	18.237 ± 1.439	1.5	13.70	3.208 ± 0.358	21.630 ± 0.166	4.022 ± 0.349	16.118 ± 1.509
	F	1.933 ± 0.224	14.610 ± 0.928	-	-	3.265 ± 0.192	25.223 ± 1.437	4.369 ± 0.343	19.819 ± 0.711
*Vespula vulgaris*	M	2.000 ± 0.202	19.250 ± 0.695	1.19	8.36	3.847 ± 0.299	35.898 ± 2.117	4.321 ± 0.214	19.611 ± 1.117
	F	2.000 ± 0.202	21.790 ± 2.595	-	-	3.680 ± 0.260	28.745 ± 0.242	4.038 ± 0.199	20.876 ± 0.864
*Vespa ducalis*	M	2.714 ± 0.200	20.143 ± 1.613	1.52 ± 0.07	15.56 ± 0.98	2.589 ± 0.247	26.375 ± 0.685	4.054 ± 0.142	19.661 ± 1.022
	F	2.754 ± 0.286	18.839 ± 2.789	1.54 ± 0.17	15.79 ± 0.63	3.461 ± 0.398	27.599 ± 1.821	4.463 ± 0.338	19.459 ± 0.577
*Vespa bicolor*	M	2.321 ± 0.159	22.018 ± 1.322	1.69	17.41	2.81 ± 0.86	32.21 ± 7.31	4.60 ± 0.26	21.23 ± 0.61
	F	2.529 ± 0.266	23.079 ± 1.814	-	-	3.552 ± 0.459	30.900 ± 1.348	5.230 ± 0.446	25.936 ± 1.634
*Vespa velutina* var. *nigrithorax*	M	2.373 ± 0.151	22.012 ± 0.841	1.45	17.42	2.589 ± 0.247	26.375 ± 0.685	4.054 ± 0.142	19.661 ± 1.022
	F	2.343 ± 0.199	16.986 ± 1.700	1.44 ± 0.09	15.08 ± 1.15	3.461 ± 0.398	27.599 ± 1.821	4.463 ± 0.338	19.459 ± 0.577
*Vespa mocsaryana*	M	2.577 ± 0.218	20.236 ± 1.35	1.32 ± 0.14	13.20 ± 1.67	3.999 ± 0.583	30.281 ± 3.312	4.856 ± 0.496	20.253 ± 0.964
	F	2.064 ± 0.192	21.978 ± 1.576	1.44 ± 0.14	15.66 ± 0.90	3.856 ± 0.266	30.481 ± 1.966	4.427 ± 1.006	22.761 ± 2.036
*P* *rovespa* *barthelemyi*	M	2.322 ± 0.096	21.730 ± 1.804	1.5 ± 0.23	15 ± 1.03	2.85 ± 0.30	30.51 ± 2.46	4.11 ± 0.35	24.06 ± 1.39
	F	2.231 ± 0.163	25.607 ± 2.315	1.56 ± 0.19	16.87 ± 1.56	3.584 ± 0.206	32.694 ± 1.280	4.671 ± 0.301	25.043 ± 0.726

Notes: M, male. F, female. The size is presented as mean ± standard deviation. The “-” marks that we have not found this sensilla. The unit is “μm”.

**Table 3:** The size of SAG, SCF, SB, PO, and SCA on the antenna (*n* = 8).

	Sensilla	SAG-I		SCF		SB		PO	SCA
Species		Wall Diameter	Length	Wall Diameter	Length	Basal Socket Diameter	Length	Inner Diameter	Bulbous Diameter
*Vespula structor*	M	3.28 ± 0.20	6.58 ± 0.70	10.357 ± 0.587	9.90 ± 1.64	2.52 ± 0.23	12.02 ± 2.85	1.856 ± 0.275	1.57
	F	3.276 ± 0.510	5.229 ± 0.720	8.250 ± 0.473	9.32 ± 1.17	2.22 ± 0.15	12.58 ± 2.20	1.796 ± 0.230	1.39
*Dolichovespula intermedia*	M	3.469 ± 0.078	5.400 ± 1.055	7.620 ± 1.043	9.31 ± 0.70	2.18 ± 0.17	9.12 ± 1.80	2.506 ± 0.163	1.366 ± 0.115
	F	4.40 ± 0.28	8.036 ± 0.367	13.406 ± 0.951	9.09 ± 0.37	2.43 ± 0.38	8.33 ± 1.38	2.337 ± 2.506	1.472
*Vespula vulgaris*	M	4.090 ± 0.404	8.0500 ± 0.792	12.409 ± 0.954	9.544 ± 1.813	2.94 ± 0.48	8.83 ± 1.39	2.025 ± 0.472	1.866 ± 0.223
	F	4.027 ± 0.404	9.386 ± 0.792	10.933 ± 0.987	10.933 ± 0.49	3.13 ± 0.38	9.13 ± 1.38	1.840 ± 0.203	1.199 ± 0.115
*D. flora*	M	4.295 ± 0.227	11.162 ± 0.6	7.935 ± 0.833	10.68 ± 0.48	3.05 ± 0.06	13.64 ± 1.04	1.902 ± 0.194	-
	F	4.016 ± 0.205	8.828 ± 0.782	9.200 ± 0.740	10.94 ± 0.85	3.24 ± 0.34	11.30 ± 1.48	2.41 ± 0.08	-
*Vespa ducalis*	M	4.49 ± 0.67	6.16 ± 1.34	9.401 ± 0.696	11.42 ± 0.95	2.93 ± 0.28	9.50 ± 1.05	-	-
	F	3.880 ± 0.246	9.463 ± 1.045	10.278 ± 0.743	11.25 ± 0.76	3.22 ± 0.24	10.60 ± 1.53	2.208 ± 0.177	-
*Vespa bicolor*	M			22.018 ± 1.322	5.71	3.49 ± 0.48	8.83 ± 0.14	2.421 ± 0.396	1.938 ± 0.088
	F	5.580 ± 0.308	8.323 ± 0.721	5.580 ± 0.308	9.78 ± 0.63	2.67 ± 0.18	8.80 ± 0.77	2.238 ± 0.338	1.970 ± 0.174
*Vespa velutina* var. *nigrithorax*	M	-	-	8.89 ± 0.16	10.22 ± 0.02	-	-	2.141 ± 0.374	1.35
	F	5.19 ± 0.17	5.55 ± 1.17	8.893 ± 1.118	11.37 ± 1.14	2.68 ± 0.21	10.57 ± 2.09	0.983	-
*Vespa mocsaryana*	M	4.316 ± 0.484	7.767 ± 2.504	10.162 ± 0.295	10.62 ± 0.58	2.73 ± 0.21	9.20 ± 1.76	3.06	-
	F	3.82 ± 0.95	4.37 ± 1.54	7.78 ± 0.74	10.36 ± 1.29	2.84 ± 0.24	10.90 ± 2.75	3.207 ± 0.188	1.83
*P* *rovespa* *barthelemyi*	M	-	-	9.872 ± 0.412	10.48 ± 1.33	3.04 ± 0.07	10.88 ± 2.85	3.791 ± 0.253	1.956 ± 0.365
	F	4.92 ± 0.80	7.38 ± 0.83	10.914 ± 0.370	12.47 ± 1.65	3.26 ± 0.16	10.52 ± 2.41	3.763 ± 0.253	1.288 ± 0.148

Notes: M, male. F, female. The size is presented as mean ± standard deviation. The “-” marks that we have not found these sensilla. The unit is “μm”.

**Table 4:** The number of tyloids on each flagellomere(F) in the different species.

	Species	*Vespula structor*	*Vespula vulgaris*	*Dolichovespula intermedia*	*D. flora*	*Vespaducalis*	*Vespa bicolor*	*Vespa velutina* v. *nigrithorax*	*Vespa mocsaryana*	*Provespa barthelemyi*
F										
F1		0	0	0	1	0	1	1	2	1
F2		0	0	0	2	1	2	2	2	2
F3		0	0	0	2	1	2	2	2	2
F4		0	0	0	2	1	2	2	2	2
F5		0	0	0	2	2	2	2	2	2
F6		0	0	0	2	2	2	2	2	2
F7		0	0	0	2	2	2	2	2	2
F8		0	0	2	2	2	2	2	2	2
F9		0	0	2	2	2	2	2	2	2
F10		0	0	2	2	2	2	2	2	2
F11		0	0	1	2	2	2	2	2	2
C/S		-	-	S	C	S	S	S	S	C

Notes: The “C/S” marks that two tyloids on each flagellomere (F) are separated or connected: C, connected; S, separated.

## Data Availability

The data presented in this study are available in the article.

## Supplementary Materials

The following supporting information can be downloaded at https://www.mdpi.com/article/10.3390/insects15060448/s1, Table S1: Antennal characters selected and coded for matrix (Description of the character state); Table S2: Matrix characters of antennae used for phylogenetic tree.
