# Supplementary material for: Comparative SEM Study of Sensilla and Tyloid Structures in the Antennae of Vespinae (Hymenoptera: Vespidae)
Source: Insects. 2024 Jun 13;15(6):448. doi: 10.3390/insects15060448 (PMC11203544; doi:10.3390/insects15060448)
Supplement: Supplementary file 1 [file insects-15-00448-s001.zip › insects-2989936-supplementary.pdf]

Supplement Table S1. Antennal characters selected and coded for matrix (Description of the character state)

Number	Character	Code character description
0	sculpture of ST-I	(1) spiricle (2) diagonal (3) longitudinal
1	length of ST-I	(1) < 16 μm (2) 16-20 μm (3) 20-25 μm (4) >25 μm
2	basal diameter of ST-I	(1) 1.6-2.0 μm (2) 2.0-2.4 μm (3) 2.4-2.8 μm (4) >2.8 μm
3	ST-II	(0) without (1) with
4	curve of ST-II	(1) bending nearly 90° (2) slightly curve (3) straight
5	difference between SP-I and_II	(1) clear (2) not clear
6	main shape of SP-I	(1) smooth (2) only concave (3) concave with carina (4) protruding
7	length of SP-I	(0) very rare without being measured (1) 18-23 μm (2) 23-24 μm (3)24-28 μm (4) >28 μm
8	Width of SP-I	(0) very rare without being measured (1) 2.5-3.0 μm (2) 3.0-3.5μm (3)3.5-4.0 μm (4) >4.0 μm
9	main shape of SP-II	(1) Smooth (2) only concave (3) concave with carina (4) protruding
10	length of SP-II	(0) very rare without being measured (1) 16-18 μm (2)18-20 μm (3)20-23 μm (4) >23 μm
11	Width of SP-II	(0) very rare without being measured (1) < 4.0 μm (2) 4.0-4.5μm (3)4.5-5.0 μm (4) >5.0 μm
12	SCF-I	(0) without (1) with
13	main shape of SCF-I	(1) central vertebral part higher than the surrounding part (2) central part at the same level as its surrounding part
14	SCF-II	(0) without (1) with
15	Open on the top of SCF-II	(0) without (1) small open （2） big open
16	Mainly shape of SCF-II	(1) central vertebral part higher than the surrounding part (2) central part at the same level as its surrounding part
17	wall diameter of SCF-II	(0) without (1) very rare without being measured (2) 5-7 μm (3) 7-9 μm (4) 9-11 μm
18	SCH-I	(0) without (1) with
19	Middle character of SCH-I	(1) with bottom (2) bottom blocked by the short walls.
20	SCH-II	(0) without (1) with
21	Middle character of SCH-II	(1) with bottom (2) bottom blocked by the short walls.
22	Open on the top of SCF-II	(0) without (1) small open (2) big open
23	SAG-I	(0) without (1) with
24	shape of SAG-I	(1) high wall (2) short wall
25	SAG-II	(0) without (1) with
26	shape of SAG-II	(1) high wall (2) short wall
27	SB-I	(0) without (1) with
28	SB-II	(0) without (1) with
29	SA	(0) without (1) with
30	SCO-I	(0) without (1) with
31	SCO-II	(0) without (1) with
32	SCA	(0) without (1) with
33	shape of SCA	(1) rounded (2) irregular rounded
34	button of SCA	(0) without SCA (1) very rare without being measured (2) 1.2-1. 4 μm (3) 1.4-1.7μm (4) >1.7 μm
35	diameter circle of SCA	(0) without SCA (1) very rare without being measured (2) 6-8 μm (3) 8-10 μm (4) >10 μm
36	SPC	(0) without (1) with

Supplement Table S2. Matrix characters of antennae used for phylogenetic tree

Character number	0	1	2	3	4	5	6	7	8	9	10	11	12	13	14	15	16	17	18	19	20	21	22	23	24	25	26	27	28	29	30	31	32	33	34	35	36
Vespa mocsaryana	2	3	2	1	1	1	1	4	3	1	3	2	0	0	0	0	0	2	1	1	1	1	1	1	2	0	0	1	0	1	1	1	1	2	1	1	0
Vesp velutina var. *nigrithorax*	2	2	2	1	1	1	2	3	2	2	1	2	0	0	1	1	2	3	1	2	0	0	0	1	1	1	2	1	0	1	0	0	1	1	2	1	0
Vespa bicolor	2	3	3	1	2	1	4	2	1	1	4	4	0	0	0	0	0	0	1	1	1	1	1	1	2	0	0	1	0	1	0	0	1	2	4	4	0
Vespa ducalis	2	2	3	1	2	1	2	3	2	2	2	2	1	2	1	1	2	4	0	0	0	0	0	1	2	1	1	1	1	0	0	0	0	0	0	0	0
Dolichovespula flora	2	1	1	1	2	1	1	3	1	1	2	2	1	1	1	1	1	4	1	2	1	1	1	1	2	1	2	1	0	0	0	0	0	0	0	0	0
Dolichovespula intermidia	2	1	1	1	2	1	1	3	1	1	2	2	0	0	0	0	0	0	0	0	1	1	1	1	2	0	0	1	1	0	0	0	1	2	3	3	0
Vespula vulgaris	2	3	1	0	2	1	1	4	2	1	3	2	0	0	0	0	0	0	1	1	0	0	0	1	2	0	0	0	0	1	0	0	0	0	0	0	0
Vespula structor	2	1	1	1	3	1	1	3	1	1	1	1	1	2	1	2	2	3	0	0	1	2	1	1	2	1	2	1	1	1	0	0	1	1	1	1	0
Parapolybia varia	2	3	3	1	2	2	3	2	3	3	2	2	0	0	1	1	1	3	0	0	0	0	0	1	2	1	1	1	1	1	0	0	0	0	0	0	0
Eustenogaster micans	2	4	4	0	0	1	3	4	2	3	3	1	0	0	0	0	0	0	0	0	0	0	0	0	0	0	0	1	0	0	1	0	0	0	0	0	1
Rhynchium quinquecincutum	2	2	3	0	0	1	1	4	4	1	3	4	1	1	1	1	1	3	0	0	0	0	0	1	2	0	0	0	1	1	0	0	1	1	3	2	0
Polistes_megei	2	3	4	1	2	2	3	2	3	3	1	2	0	0	1	1	1	4	0	0	1	1	1	1	1	1	2	1	1	1	0	0	1	1	1	1	0
